# Oxytocin specifically participates in social memory encoding by exciting stellate neurons in the lateral entorhinal cortex

**DOI:** 10.3389/fnbeh.2026.1834610

**Published:** 2026-08-20

**Authors:** Jinquan Wang, Feng Cai, Qi Zhu, Zijian Song, Hao Zhang, Haiyan Wang, Jiaxiang Duan, Hua Huang, Yi Xu

**Affiliations:** 1 Department of Anesthesiology, Chongqing Liangjiang New Area Traditional Chinese Medicine Hospital, Chongqing, China; 2 927th Hospital of Joint Logistics Support Force, PLA, Pu’er, Yunnan, China; 3 Department of Anesthesia, General Hospital of Tibet Military Command, Lhasa, Tibet, China; 4 Department of Anesthesia, Southwest Hospital, Army Medical University, Chongqing, China

**Keywords:** lateral entorhinal cortex, oxytocin, social memory, spatial memory, stellate neurons

## Abstract

**Introduction:**

Oxytocin (OXT) has emerged as a key neuromodulator of social cognition, yet the precise neural circuits underlying its role in social memory remain poorly defined. In this study, we identify a specific mechanism by which OXT regulates social memory encoding through the lateral entorhinal cortex (LEC), a critical gateway to the hippocampus.

**Methods:**

Using whole-cell patch-clamp recordings in mouse brain slices, we examined the effects of OXT on stellate neurons in the superficial layers of the LEC. Behavioral analyses were performed to determine the role of intra-LEC OXT signaling in the encoding and retrieval of social memory. Pharmacological blockade of OXTRs in the LEC was used to assess its effects on social recognition memory and spatial memory performance.

**Results:**

OXT excited stellate neurons in the superficial layers of the LEC via activation of oxytocin receptors (OXTRs). This excitation was mediated by membrane depolarization and enhanced excitatory synaptic transmission, as evidenced by an increased amplitude of miniature excitatory postsynaptic currents. Intra-LEC OXT signaling was essential for the encoding, but not the retrieval, of social memory. Pharmacological blockade of OXTRs in the LEC selectively impaired social recognition memory without affecting spatial memory performance. Furthermore, inhibition of OXTRs during the recall phase did not disrupt memory expression, indicating a specific role in memory formation.

**Discussion:**

These findings establish the LEC as a critical node for OXT-dependent modulation of social memory and suggest that OXT enhances the flow of social information to the hippocampus via direct excitation of LEC stellate neurons. This circuit-specific action provides a mechanistic basis for the selective influence of OXT on social cognition and highlights the LEC–hippocampal pathway as a potential target for therapeutic intervention in social memory deficits associated with neuropsychiatric disorders.

## Introduction

Oxytocin (OXT), a neuropeptide hormone historically recognized for its pivotal roles in lactation and parturition, has emerged as a critical modulator of complex social behaviors. Currently, advances in neurobiology have expanded our understanding of OXT’s pleiotropic functions, revealing its involvement in regulating anxiety (Li et al., 2016), fear extinction (Arellano Perez et al., 2024), and reward processing (Hu et al., 2015). Notably, OXT exhibits a robust association with social memory, a cognitive process essential for recognizing conspecifics, maintaining social bonds, and navigating complex social environments (Cymerblit-Sabba et al., 2023; Liu et al., 2026; Liu et al., 2025; Lukas et al., 2013). This link has sparked intense interest in OXT as a potential therapeutic target for neurodevelopmental and psychiatric disorders characterized by social deficits, such as autism spectrum disorder (ASD) (Higashida et al., 2012).

Several studies have provided compelling evidence for OXT’s role in social memory enhancement. Rodent models have demonstrated that OXT administration strengthens social recognition memory, while human trials using intranasal OXT have reported improvements in social cognition and memory formation in individuals with ASD (Fooladi et al., 2025; John and Jaeggi, 2021; Liu et al., 2026; Rigney et al., 2022). However, the precise neural mechanisms by which OXT modulates social memory remain incompletely understood. A growing body of literature suggests that OXT acts through specific neural circuits, including the medial prefrontal cortex, amygdala, and hippocampus (Hu et al., 2015; Cymerblit-Sabba et al., 2023; Liu et al., 2026; Liu et al., 2025; Lukas et al., 2013).

Recent studies have shown that the lateral entorhinal cortex (LEC) plays a crucial role in memory formation, especially in social memory formation. The LEC is regarded as the gateway to the hippocampus with its superficial layers (layers 2 and 3) receiving multimodal non-spatial contextual information and project it to the dentate gyrus and CA2 regions of the hippocampus, participating in social memory formation. Conversely, its deep layers receive inputs from CA1 and the subiculum of the hippocampus, and project to the medial prefrontal cortex (Chen et al., 2018; He et al., 2016; Valenti and Grace, 2009; Van Cauter et al., 2013). In the superficial layers of the LEC, stellate neurons are predominant, which project to the hippocampus via the perforant path, thus participating in memory formation (Alessi et al., 2016; Hargus et al., 2012; Richter et al., 2000). Inhibiting the LEC-CA2 neural circuit can lead to severe impairment of social memory (Lopez-Rojas et al., 2022). Although the LEC plays an important role in social memory, it remains unclear how it is regulated by OXT.

In the present study, we found that OXT can excite stellate neurons in the LEC and enhance the excitatory synaptic inputs to these neurons. In behavioral studies, it was found that OXT in the LEC specifically participates in the encoding of social memory but has no obvious effect on the retrieval of social memory. Moreover, blocking OXT receptors (OXTRs) in the LEC not affect spatial memory. These findings further reveal the mechanisms by which OXT regulates social memory.

## Methods

All procedures involving C57BL/6 male mice (8–12 weeks) were approved by the Animal Care and Use Committee of Department of Anesthesia, Southwest Hospital, Army Medical University. The rationale behind our selection of 8–12 week-old C57BL/6 mice is well grounded in established literature (Lopez-Rojas et al., 2022; Watarai et al., 2025), which shows that C57BL/6 mice exhibit stable social memory performance. Of note, C57BL/6 mice may develop progressive hearing decline and retinal degeneration after 3 months of age. In the present study, all experimental animals were strictly maintained within the 3-month age limit throughout the entire behavioral testing timeline. To eliminate potential confounding effects of the estrous cycle on the consistency of behavioral performance, we exclusively utilized male animals rather than female subjects in the present study.

### Brain slice preparation

After anesthetizing the mice with isoflurane (2%), brains were removed carefully following decapitation and submerged into ice-cold oxygenated (95% O_2_ and 5% CO_2_) artificial cerebrospinal fluid (ACSF; in mM: 124 NaCl, 3 KCl, 26 NaHCO₃, 2 MgCl₂, 2 CaCl₂, 10 glucose). Coronal brain slices (400 μm) containing the LEC were prepared using a vibrating blade microtome (Leica VT1000S, Germany) in ice-cold cutting solution (in mM: 220 sucrose, 2.5 KCl, 1.25 NaH₂PO₄, 26 NaHCO₃, 6 MgCl₂, 1 CaCl₂, 10 glucose; equilibrated with 95% O₂/5% CO₂). Slices were incubated for ≥ 30 min at 32 °C in oxygenated ACSF before recording. For experiments, slices were transferred to a submersion chamber perfused with ACSF (3 mL/min).

### Whole-cell patch-clamp recordings in LEC neurons

Recordings were performed on LEC stellate neurons (identified via infrared differential interference contrast microscopy; Leica DMI6000B). Patch pipettes (4–6 MΩ) were filled with internal solution (in mM: 125 potassium gluconate, 20 KCl, 10 HEPES, 1 EGTA, 2 MgCl₂, 4 ATP-Na₂; pH 7.3, 290 mOsm). A tight seal (1–2 GΩ) was formed by negative pressure, followed by membrane rupture via gentle suction to establish the whole-cell configuration. Signals were amplified (EPC10, HEKA Elektronik, Germany), low-pass filtered at 4 kHz, and digitized at 10 kHz. Data were analyzed offline using Pulse/PulseFit v8.74 (HEKA) and Igor Pro 5.04 (WaveMetrics). Neurons were excluded if series resistance increased by >15% from baseline or exceeded 25 MΩ during recordings.

In voltage-clamp configuration, the membrane potential of LEC stellate neurons was held at −60 mV to isolate and quantify the net inward current evoked by bath-applied OXT. After at least 6 min of stable recording, OXT was perfused for 2 min, and then washout for at least 9 min. To detect the receptor mechanism of oxytocin, we subsequently perfused 6 min of cligosiban in the same cell and then added OXT to observe its effects on membrane potential and membrane current in the presence of the cligosiban. The average values of the 1 min before pharmacological substance administration were used as baseline. The effect was often the greatest 1 min after OXT perfusion, so we used the average value 1 min after perfusion as the value for the treatment group.

To record spontaneous postsynaptic currents (sEPSCs) and miniature excitatory postsynaptic currents (mEPSCs), we continuously infused the GABA_A_R antagonist picrotoxin (PIC, 100 μM) and the sodium channel blocker tetrodotoxin (TTX, 1 μM) plus PIC (100 μM) into the ACSF, respectively. The membrane potentials were clamped at +60 mV to record the sEPSCs and mEPSCs. The events of spontaneous or miniature postsynaptic currents with rapid onset and characteristic kinetics of exponential decay were first automatically detected by the MiniAnalysis software with pre-set parameters, and then re-checked with manual verification. The average frequency and amplitude of postsynaptic currents recorded during the 1-min period before drug administration were taken as baseline values. The pharmacological substance was then perfused for 3 min, and the average value recorded in the last minute was used as the treatment group.

### Preparation of pharmacological substances

Cligosiban is a novel, brain-penetrant and highly selective antagonist targeting the OXT receptor (Wayman et al., 2018). All pharmacological substances were obtained from Sigma-Aldrich (St Louis, MO, USA: picrotoxin, dimethyl sulfoxide, tetrodotoxin), MedChemExpress LLC (cligosiban), and Tocris Cookson (Ellisville, MO, USA: OXT). Stock solutions (stored at −20 °C) were freshly diluted to working concentrations. Cligosiban and picrotoxin were dissolved in 0.5% dimethyl sulfoxide with ACSF. Unless otherwise specified, other pharmacological substances were dissolved in ACSF. Bath perfusion of OXT, cligosiban, TTX, and PIC was applied to the recording chamber throughout whole-cell patch-clamp experiments.

### Cannula guide implantation and pharmacological substance injection

Mice were anaesthetized with isoflurane (2% for induction and 1%–1.5% for maintenance). The head of the mice was first fixed on a stereotactic frame (RWD Life Technology Co., Ltd). After the scalp was surgically resected to expose the skull surface, targeted cranial holes were drilled at pre-calibrated coordinates. A custom cannula guide was then implanted above the LEC, positioned at stereotactic coordinates of −3.4 mm anteroposterior (AP), ±4.7 mm mediolateral (ML), and 2.3 mm dorsoventral (DV) relative to the bregma, with a small pedestal securing the base of the guide. The cannula guide was fixed firmly to the skull using cyanoacrylate adhesive and light-cured dental cement. A sterile dummy cannula was inserted into the inner lumen of the guide to prevent blockage and contamination during post-surgical recovery. For subsequent intracerebral pharmacological substance infusion, the dummy cannula was gently withdrawn, and an injector cannula extending exactly 0.5 mm beyond the tip of the pre-implanted guide was inserted to deliver agents directly into the targeted LEC parenchyma.

During the microinjection of pharmacological substances, we wrapped the mice with towels to prevent them from struggling, and then inserted the injection needle to complete the microinjection. To prevent the injection process from causing stress in the mice, which may interfere with their behavior, we will acclimate the mice 3 days in advance. We wrapped the mice with strips of cloth three times a day, each time for 10 min, and simulate inserting the injection needle for microinjection to reduce the struggling reaction of the mice. The pharmacological substance was administered by syringe pump (KD Scientific), and infused over 2 min with speed at 50 nL/min. The injector cannula was removed 2 min after the end of the micro-infusion to avoid pulling out the pharmacological substance and the dummy cannula was put back. Behavioral testing started 15 min after infusion. The vehicle is composed of 5% dimethyl sulfoxide diluted in ACSF. For all intra-LEC microinjections, both vehicle and the selective oxytocin receptor antagonist cligosiban (1 μM) were delivered at a total volume of 100 nL per hemisphere.

### Behavioral tests

The male C57BL/6 mice with 8–12 weeks of age were used for the behavioral tests at the dark phase, within a fixed 3-h window from 20:00 to 23:00. After surgery, animals were handled daily and accommodated to the recording arena for 1 week before starting experiments. Social memory and spatial memory tasks were performed as previously described (Lopez-Rojas et al., 2022; Oliva et al., 2020). Briefly, a subject male mouse was habituated for 10 min to a rectangular arena (40 cm × 20 cm × 20 cm) with two empty wire cups in opposite sides. After this, in an encoding phase, two novel stimulus male mice (S1 and S2) that had no prior contact with the subject male mouse was placed inside each of the cups and the subject mouse was allowed to explore the arena with the two novel mice for 10 min. The subject mouse was then isolated for 120 min and one of the stimulus male mice was exchanged for a third novel mouse (N). In the recall phase of the test, the subject mice were exposed to one of the now-familiar stimulus mice, previously encountered during the learning trial, along with the novel stimulus male mouse. Social exploration was quantified as the time spent in active exploration within 5 cm of the perimeter of the cup. We then assessed social memory with a discrimination index (DI) = (time exploring mouse N − time exploring mouse S)/(time exploring mouse N + time exploring mouse S).

During the spatial memory task, male mice first underwent habituation in an empty context (30 cm × 30 cm × 20 cm) for 10 min. On the following day, two identical objects were placed in the familiar context. During the encoding stage, mice freely explored two identical objects (5 cm in diameter) for 10 min. After 2-h interval in the home cage, a 10-min recall phase followed. In this recall phase, the spatial position of object B was changed, and mice were allowed to re-explore the two objects. Video recording was performed to record the exploration and re-exploration activities for further analyses. Spatial memory was assessed with a DI = (time exploring object B − time exploring object A)/(time exploring object B + time exploring object A).

### Statistical analysis

Data are presented as mean ± SEM (*n* = number of neurons/animals). Normality and equal variances were formally tested for each group of data. Specifically, normality testing was performed using the Shapiro–Wilk normality test. Homogeneity of variances was assessed using the Bartlett test. For data with normal distribution and equal variances, unpaired t-tests, paired t-tests or Repeated measures ANOVA by Tukey’s test for multiple-group comparisons were performed for the comparisons between groups. If data did not conform to the normal distribution, nonparametric tests were performed. *p* < 0.05 was considered significant.

## Results

### OXT changed in membrane potential and firing activity of stellate neurons in the superficial layers of the LEC

**Figure 1:**
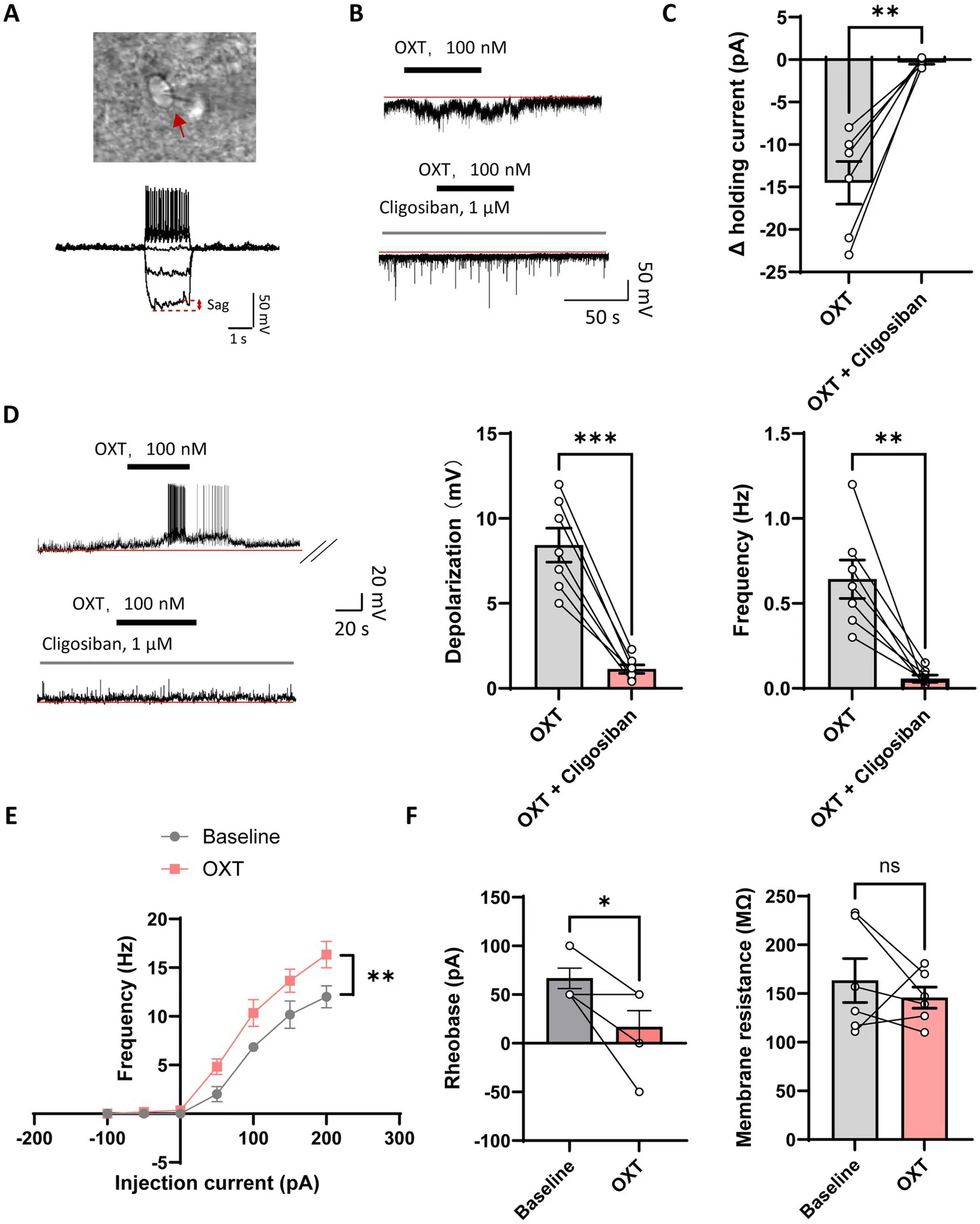
Oxytocin (OXT) induces an inward current in stellate neurons of the lateral entorhinal cortex (LEC), and causes depolarization and an increase in the firing frequency. **(A)** A recording stellate glutamatergic neuron in the LEC (top) and the corresponding electrophysiological traces (bottom). **(B)** Example holding current traces before, during and after bath application of the 100 nM OXT in the absence and presence of the cligosiban (1 μM). **(C)** The OXT-induced changes in the holding currents in the absence and presence of the cligosiban. *n* = 6 cells from 3 mice. **(D)** Left, example membrane potential traces before, during and after bath application of the 100 nM OXT in the absence and presence of the cligosiban (1 μM). The OXT-induced depolarization (middle) and increased firing rates (right) in the absence and presence of the cligosiban. *n* = 7 cells from 4 mice. **(E)** Bath application of OXT significantly increases the number of action potentials evoked by incremental step current injections in LEC stellate neurons (*n* = 7 cells from 3 mice). **(F)** Effect of the OXT on the rheobase and input resistance of the stellate neurons (*n* = 7 cells from 3 mice). **p < 0.001, ***p < 0.0001. Data are presented as mean ± SEM.

The axons of stellate neurons in the superficial layers of the EC form the perforant pathway and provide the major excitatory inputs to the hippocampus, especially the social memory-related CA2 region (Alessi et al., 2016; Lopez-Rojas et al., 2022). These neurons were identified by their morphology and electrophysiological properties (He et al., 2016). Stellate neurons exhibited oval soma with multiple primary dendrites. These neurons had notable features that distinguished them from other cells, including a profound voltage sag in response to a hyperpolarizing current (Figure 1A). Based on the electrophysiological recordings, stellate neurons exhibited a membrane capacitance of 104.46 ± 7.20 pF (*n* = 13 cells) and a resting membrane potential of −58.68 ± 0.77 mV (*n* = 13 cells) (mean ± SEM). Previous literature reports that the average input impedance of interneurons in the entorhinal cortex exceeds 200 MΩ (Liao et al., 2024), suggesting that stellate neurons have different electrophysiological characteristics.

We first carried out voltage- or current-clamp recordings to explore the effects of OXT on the excitability of stellate neurons. During voltage-clamp mode, application of OXT (100 nM) induced inward currents in the recorded stellate neurons. The OXT-induced inward currents were reversible following washout, and could be blocked by OXTR antagonist cligosiban (1 μM) (OXT: −14.5 ± 2.5 pA, OXT + cligosiban: −0.34 ± 0.20 pA, paired *t*-test, *t*_5_ = 5.66, *p* = 0.0024, Figures 1B,C). The fact that OXT induced a stable inward current suggests a OXTRs-mediated excitatory effect.

Next, we tested the effect of OXT on the membrane potential and firing of stellate neurons during current clamp. The bath application of OXT remarkably increased the firing rates (OXT: 0.64 ± 0.30 Hz, OXT + cligosiban: 0.06 ± 0.02 Hz, paired *t*-test, *t*_6_ = 4.915, *p* = 0.0027) of stellate neurons and induced a sufficiently depolarization (OXT: 8.4 ± 1.0 mV, OXT + cligosiban: 1.1 ± 0.3 mV, paired *t*-test, *t*_6_ = 8.797, *p* < 0.001) to activate these neurons (Figure 1D). The depolarization and increased firing frequency caused by OXT can be blocked by its receptor antagonist. In the presence of OXT, LEC stellate neurons generate a substantially greater number of action potentials in response to injected step current stimuli (*n* = 7 cells, Two-way Repeated Measures ANOVA, F_6, 60_ = 2.734, baseline vs. OXT: *p* = 0.0049, Figure 1E). While OXT markedly reduces the rheobase (Baseline: 66.7 ± 10.5 mV, OXT: 16.7 ± 16.7 mV, paired *t*-test, t_5_ = 3.873, *p* = 0.0117, Figure 1F) of these stellate cells, it exerts no significant modulatory effect on their input resistance (Baseline: 163.3 ± 22.5 mV, OXT: 145.7 ± 10.8 mV, paired *t*-test, t_5_ = 0.7956, *p* = 0.4624, Figure 1F).

### OXT enhanced the excitatory synaptic input of the stellate neurons

**Figure 2:**
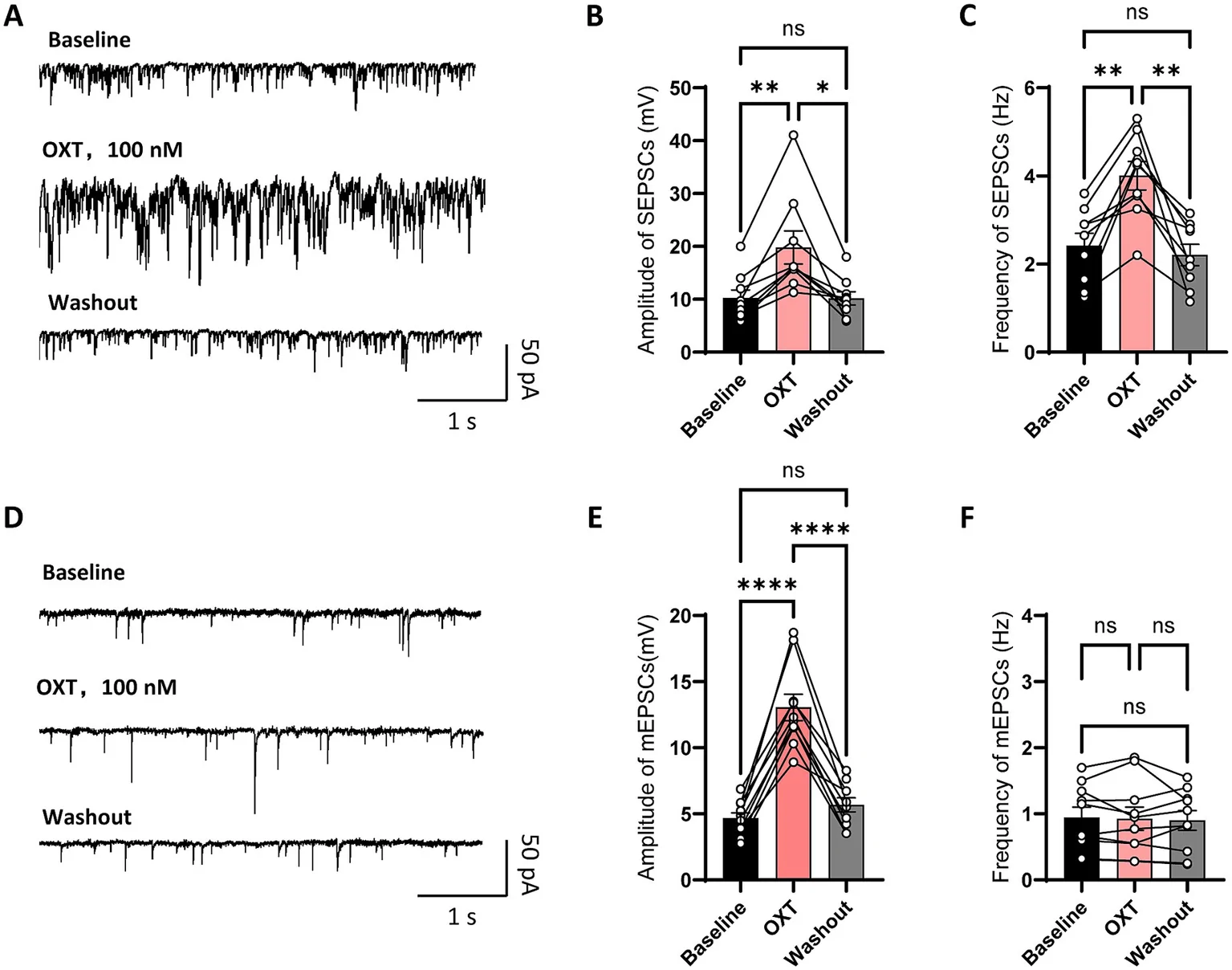
OXT enhances excitatory synaptic input. **(A)** Example traces of SEPSCs before, during and after bath application of the 100 nM OXT. **(B,C)** The changes in the amplitude **(B)** and frequency **(C)** of the SEPSCs before, during and after bath application of the OXT. *n* = 9 cells from 4 mice. **(D)** Example traces of SEPSCs before, during and after bath application of the 100 nM OXT. **(E,F)** The changes in the amplitude **(E)** and frequency **(F)** of the mEPSCs before, during and after bath application of the OXT. *n* = 10 cells from 4 mice. *p < 0.05, **p < 0.01, ***p < 0.001, ****p < 0.0001. Data are presented as mean ± SEM.

The activity of stellate neurons is also influenced by synaptic transmission. We further recorded SEPSCs in the presence of GABA_A_ receptor antagonist picrotoxin (100 μM) at a fixed potential of −60 mV. After a stable recording, bath application of OXT increased the amplitude (baseline: 10.27 ± 1.49 pA, OXT: 19.81 ± 3.12 pA, Washout: 10.20 ± 1.24 pA, One-way Repeated measures ANOVA, F_2, 16_ = 15.53, *p* = 0.0029, Figures 2A,B) and frequency (baseline: 2.42 ± 0.28 Hz, OXT: 4.01 ± 0.32 Hz, Washout: 2.21 ± 0.24 Hz, One-way Repeated measures ANOVA, F_2, 16_ = 13.33, *p* = 0.0008, Figures 2A,C) of the SEPSCs, indicating that OXT enhances the glutamatergic input in the superficial layers of the LEC.

Next, we further recorded mEPSCs in the presence tetrodotoxin (1 μM) to block voltage-gated Na^+^ channels. It has been reported that an alteration in the amplitude of mEPSCs reflects a postsynaptic mechanism, whereas a change in the frequency of these currents signifies a change in the presynaptic glutamate release (He et al., 2016). Application of OXT did not influence the frequency (baseline: 0.95 ± 0.16 Hz, OXT: 0.93 ± 0.18 Hz, Washout: 0.90 ± 0.15 Hz, One-way Repeated measures ANOVA, F_2, 18_ = 0.227, *p* = 0.7469, Figures 2D,F), but enhanced the amplitude (baseline: 4.67 ± 0.41 pA, OXT: 13.04 ± 1.00 pA, Washout: 5.68 ± 0.53 pA, One-way Repeated measures ANOVA, F_2, 18_ = 71.39, *p* < 0.0001, Figures 2D,F) of the mEPSCs. Together, these data indicate that OXT may enhance the glutamatergic transmission via influencing the postsynaptic receptors.

### Blocking OXTRs in the LEC impaired the encoding, but not the recall, of the social memory

**Figure 3:**
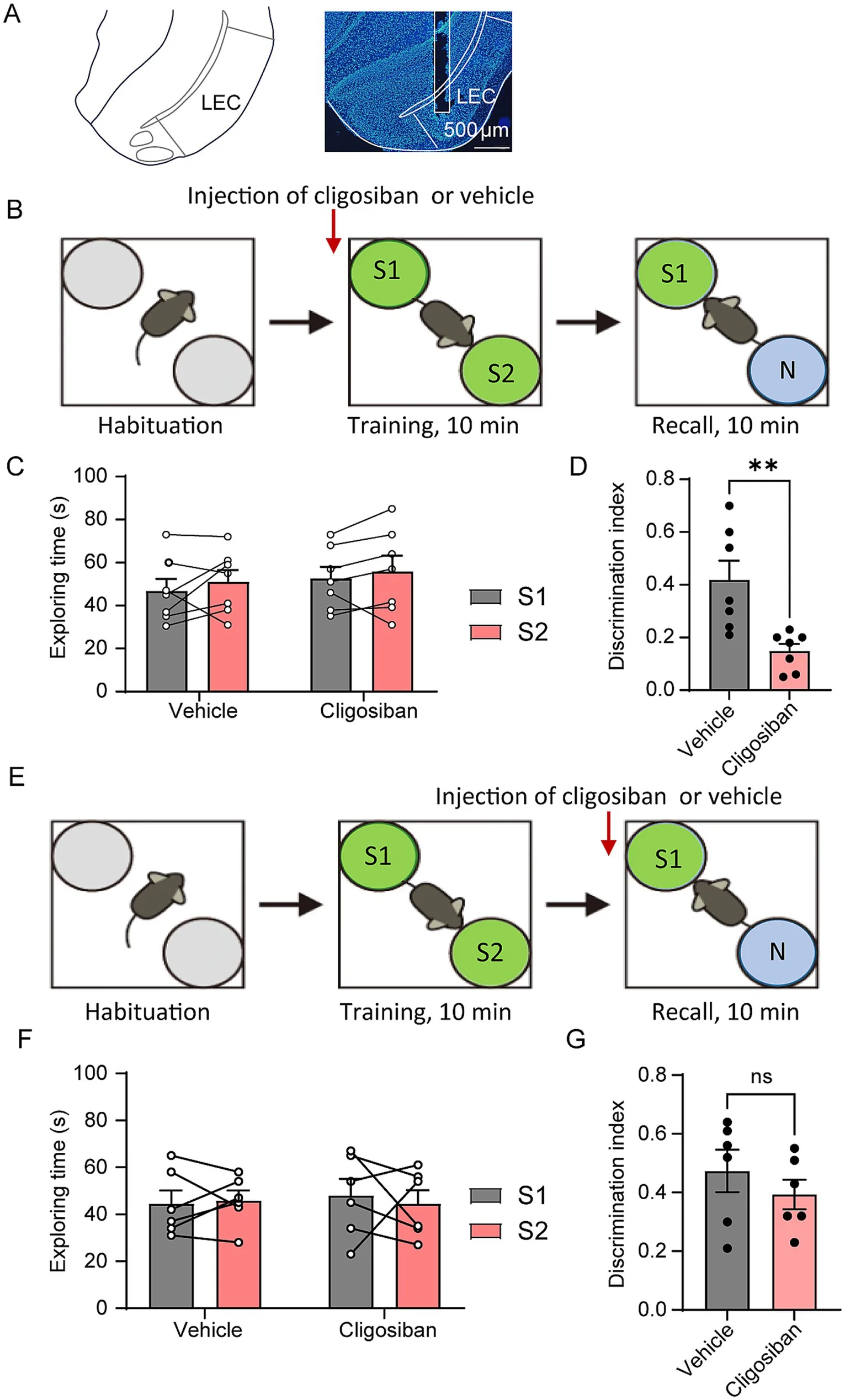
OXT signaling in the LEC is required for the encoding, but not the recall, of the social memory. Illustration of implantation of cannulae in the LEC **(A)**. Schema of the social memory task **(B)**. The exploring time of the novel mouse S1 and S2 during the encoding phase with the injection of vehicle and cligosiban (1 μM) before the encoding phase **(C)**. *N* = 7 mice for each group. Effect of the vehicle and cligosiban on discriminative index during the recall phase **(D)**. *N* = 7 mice for each group. Schema of the social memory task with the injection of vehicle and cligosiban (1 μM) before the recall phase **(E)**. The exploring time of the novel mouse S1 and S2 during the encoding phase with injection of vehicle and cligosiban (1 μM) before the recall phase **(F)**. *N* = 6 mice for each group. Effect of the vehicle and cligosiban on (1 μM) discriminative index during the recall phase **(G)**. *N* = 6 mice for each group. **p < 0.01. NS, no significance. Data are presented as mean ± SEM.

To investigate the role the LEC OXT signaling in social memory, animals were engaged in an open arena, two-choice social memory task. In the encoding phase of this task, a subject mouse was allowed to explore for 10 min in which two novel stimulus mice were presented in wire cup cages in opposite corners of the arena. The subject mouse was then placed in its home cage for 120 min, after which a memory recall test was performed, in which the subject mouse explored for 10 min the same arena containing one of the two original stimulus mice and one completely novel mouse. Social memory was assessed by the preference of the subject mouse to explore the novel mouse compared with the original, now-familiar stimulus mouse (Figures 3A,B).

We first examined the effects of blocking the OXTRs in the LEC during encoding phase (Figure 3B). As expected, control mice with injection of vehicle in the LEC spent significantly more time interacting with a novel animal compared with the now-familiar mouse in the memory recall trial. However, injection of the OXTR antagonist in the LEC produced a significant impairment in social memory performance, based on the similar amount of time such mice spent exploring the novel and now-familiar mouse during the recall trial (*n* = 7 for each group, unpaired *t*-test, *t*_12_ = 3.473, *p* = 0.0046, Figure 3D). Whether it is the control group animals or the animals with inhibited OXTRs in the LEC area, during the encoding stage, there was no difference in the time spent exploring the S1 and S2 animals (Vehicle: *n* = 7; Cligosiban: *n* = 7; main effect of pharmacological treatment: F_1, 12_ = 0.4341, *p* = 0.5224; main effect of novel stimulus mice: F_1, 12_ = 1.630, *p* = 0.2259; interaction: F_1, 12_ = 0.02675, *p* = 0.8728; two-way repeated-measures ANOVA, Figure 3C), indicating that the social memory encoding impairment caused by this inhibition of OXTRs is not due to differences in the exploration motivation of different animals.

Additionally, in another separate group of animals, we administered an OXTR antagonist to the LEC before recalling social memory to investigate its effect on social memory retrieval (Figure 3E). During the encoding stage, there was no difference in the time spent exploring the S1 and S2 animals (Vehicle: *n* = 6; Cligosiban: *n* = 6; main effect of pharmacological treatment: F_1, 10_ = 0.02722, *p* = 0.8723; main effect of novel stimulus mice: F_1, 10_ = 0.04923, *p* = 0.8289; interaction: F_1, 10_ = 0.2450, *p* = 0.6313; two-way repeated-measures ANOVA; Figure 3F). Under this condition, both the control mice and the mice with injection of OXTR antagonist in the LEC spent significantly more time interacting with a novel animal compared with the familiar mouse in the memory recall trial (*n* = 6 for each group, unpaired *t*-test, *t*_10_ = 0.9088, *p* = 0.3848, Figure 3G), implying the blocking the OXTR during recall phase did not affect the social memory recall.

### Blocking OXTRs in the LEC did not affect the encoding and recall of the spatial memory

**Figure 4:**
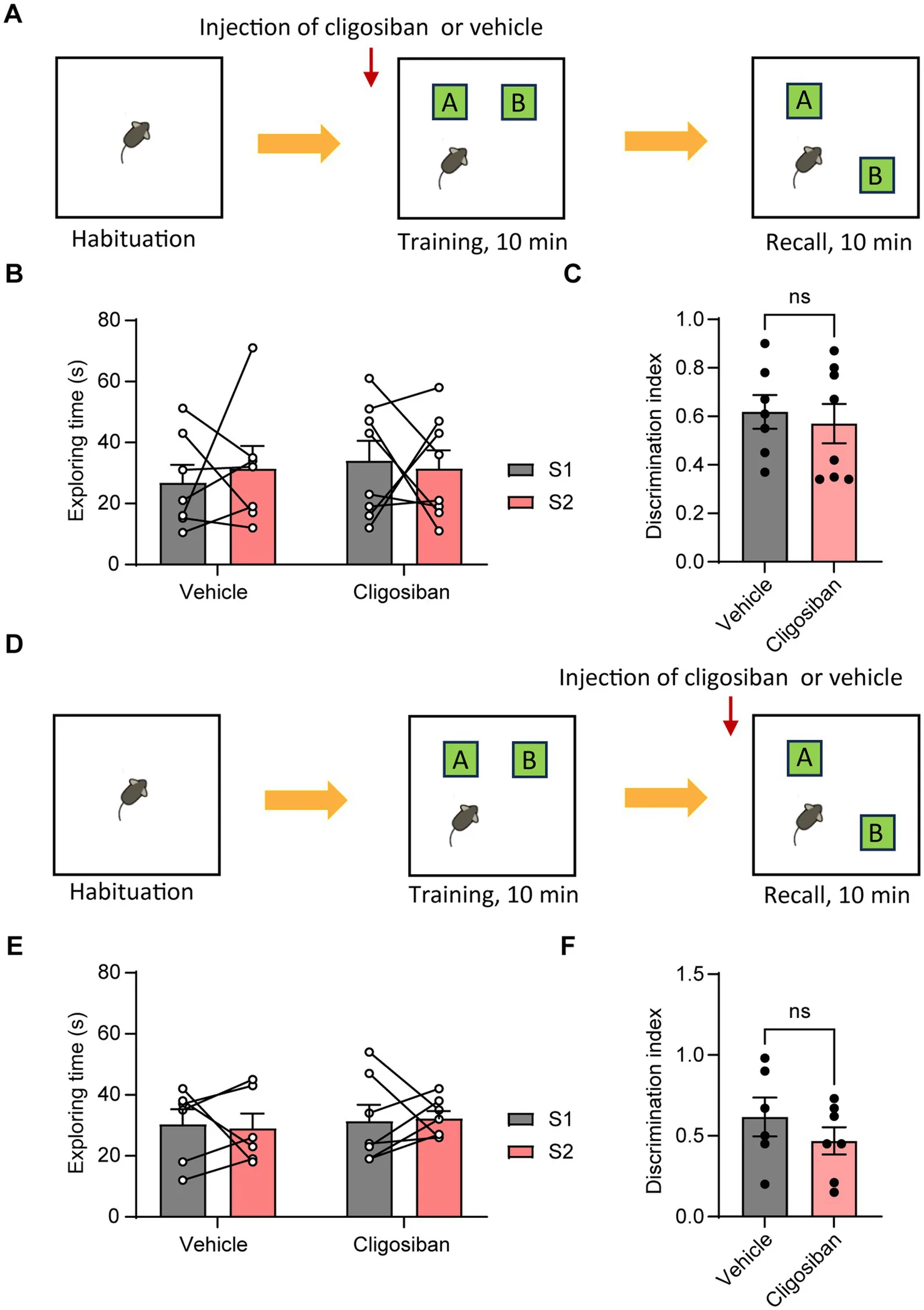
Blockade of OXTR in the LEC does not affect the spatial memory. **(A)** Schema of the spatial memory task. **(B)** The exploring time of the object A and B during the encoding phase with the injection of vehicle (*n* = 7) and 1 μM cligosiban (*n* = 8) before the encoding phase. **(C)** Effect of the vehicle and cligosiban on discriminative index during the recall phase. **(D)** Schema of the spatial memory task with the injection of vehicle and cligosiban before the recall phase. **(E)** The exploring time of the object A and B during the encoding phase with injection of vehicle (*n* = 6) and 1 μM cligosiban (*n* = 7) before the recall phase. **(F)** Effect of the vehicle and cligosiban on discriminative index for object A and B during the recall phase. Ns, no significance. Data are presented as mean ± SEM.

To confirm that OXT signaling in the LEC was specific to the social memory, we conducted another spatial memory task. In this task, mouse was allowed to explore for 10 min a square arena in which two novel objects. After 120 min, object B was removed to a novel location (Figure 4A). Local injection of the OXTR antagonist in the LEC before encoding phase did not affect the time spent in exploring location of object A and object B during the encoding phase (Vehicle: *n* = 7; Cligosiban: *n* = 8; main effect of pharmacological treatment: F_1, 13_ = 0.3297, *p* = 0.5757; main effect of objecct: F_1, 13_ = 0.0244, *p* = 0.8782; interaction: F_1, 13_ = 0.2821, *p* = 0.6043; two-way repeated-measures ANOVA; Figure 4B). During the following recall phase, the mice still showed a preference to recognize the changed spatial position of object B, indicating blocking OXTRs did not influence the spatial memory encoding (Vehicle: *n* = 7 mice, Cligosiban: *n* = 8 mice, unpaired *t*-test, *t*_13_ = 0.4472, *p* = 0.6621, Figure 4C).

Additionally, we locally injected the OXTR antagonist in the LEC before the recall phase in another separate group of animals (Figure 4D). Under this condition, the mice with injection of OXTR antagonist still showed a preference to recognize the object B (Vehicle: *n* = 6 mice, Cligosiban: *n* = 7 mice, unpaired *t*-test, *t*_11_ = 1.033, *p* = 0.3240, Figure 4F). Furthermore, whether it is the control group animals or the animals with inhibited OXTRs in the LEC area, there was no difference in the time spent in exploring location of object A and object B during encoding phase (Vehicle: *n* = 6; Cligosiban: *n* = 7; main effect of pharmacological treatment: F_1, 11_ = 0.1981, *p* = 0.6649; main effect of object: F_1, 11_ = 0.0034, *p* = 0.9545; interaction: F_1, 11_ = 0.0722, *p* = 0.7932; two-way repeated-measures ANOVA; Figure 4E). Altogether, these data indicate that OXT signaling in the LEC is not required for the encoding and recall of the spatial memory.

## Discussion

The medial entorhinal cortex (MEC) has long been recognized as a critical hub for spatial memory, with its grid cells, border cells, and head-direction cells forming the foundational cognitive map that enables navigation and spatial representation (Kropff et al., 2015; Tukker et al., 2022). However, the functional role of the LEC has remained relatively understudied until recent years. Emerging evidence from both animal models and human neuroimaging studies has begun to unravel the LEC’s unique contributions, revealing its involvement in processing temporal sequences, and non-spatial episodic memories (Tsao et al., 2018; Kanter et al., 2025; Issa et al., 2024; Kuhn et al., 2024; Tozzi et al., 2024). Notably, the LEC has been implicated in olfactory associative learning, where it links sensory inputs to contextual memories (Liu, 2020).

OXT, a neuropeptide best known for its roles in social bonding, maternal behavior, and stress regulation, has been extensively studied in the context of social memory (Liu et al., 2025; Lukas et al., 2013). However, its precise mechanisms of action within the LEC have remained elusive. Our study addresses this gap by identifying that OXT exerts an excitatory effect on LEC stellate neurons, which are the primary projection neurons of the LEC’s superficial layers. The observed effects were related to OXTR activation upon binding to OXTRs, OXT activates the Gq/11 protein-coupled signaling pathway, leading to the activation of phospholipase C and elevation of Ca^2+^ in the cytoplasm (Menon and Neumann, 2023). This enzymatic cascade has two key consequences: first, it closes voltage-gated potassium channels, leading to membrane depolarization and enhanced neuronal excitability (Tateyama and Kubo, 2023; Li et al., 2017; Schiekel et al., 2013); second, it may modulate postsynaptic ionotropic glutamate receptors, thereby strengthening excitatory synaptic transmission (Marron et al., 2025). These cellular mechanisms collectively enhance the ability of LEC stellate neurons to integrate and transmit multisensory information to the hippocampus, providing a mechanistic basis for OXT’s role in promoting social memory formation.

Although our data support a possible postsynaptic mechanism for OXT-mediated enhancement of glutamatergic transmission in stellate neurons, we note that increased mEPSC amplitude could theoretically reflect presynaptic changes in quantal content under specific conditions (e.g., altered vesicular release probability). Crucially, however, the concomitant elevation in sEPSC frequency observed in our study suggests potential presynaptic involvement. Future experiments should include detection of whether oxytocin receptors are specifically expressed in neuronal cell bodies to enable further verification. The functional connectivity between the LEC and hippocampus relies on precise neural oscillations that facilitate high-fidelity information transfer. Theta and gamma oscillations have been shown to play a critical role in coordinating neural activity between brain regions during memory encoding. For example, fast gamma oscillations (100–150 Hz) synchronize MEC and dentate gyrus activity during spatial learning, while slow gamma oscillations (30–50 Hz) coordinate LEC and dentate gyrus activity during object learning (Fernández-Ruiz et al., 2021). OXT’s modulation of LEC stellate neuron activity may functionally enhance oscillatory synchrony and optimize entorhinal-hippocampal information transfer, ultimately facilitating social memory encoding.

The LEC communicates with the hippocampal CA2 region via two distinct pathways: an indirect pathway through the dentate gyrus and a direct monosynaptic pathway from the LEC’s superficial layers to CA2. Previous research has demonstrated that inhibiting the dorsal dentate gyrus does not impair social memory encoding, suggesting that the indirect pathway is not critical for this process. In contrast, blocking the direct LEC-CA2 pathway severely disrupts social memory, indicating that this direct projection is essential for encoding social information (Lopez-Rojas et al., 2022). Our finding that OXT excites LEC superficial stellate neurons, the primary source of the direct LEC-CA2 projection, strongly suggests that OXT’s facilitatory effect on social memory is mediated by enhancing activity in this direct pathway. This circuit-specific mechanism ensures that OXT selectively modulates social memory processes without interfering with other memory domains, such as spatial memory. The specificity of this pathway also highlights the importance of dissecting neural circuits at the level of projection targets, as different pathways from the same brain region can subserve distinct behavioral functions.

Consistent with the LEC’s specialization in non-spatial memory (Fernández-Ruiz et al., 2021), our study further demonstrates that blocking OXTRs in the LEC does not affect spatial memory performance. This result aligns with prior work showing that the MEC, not the LEC, is critical for spatial memory, and that the MEC-CA2 projection is weaker and insufficient to support social memory (Lopez-Rojas et al., 2022). These findings collectively indicate that OXT’s regulation of social memory is highly circuit-specific, targeting the LEC-CA2 pathway rather than broader spatial memory networks. This specificity is biologically meaningful, as it allows for independent modulation of social and spatial memory processes, which are often engaged in distinct behavioral contexts. For example, during social interactions, the LEC-CA2 pathway may be prioritized to encode social cues, while during navigation, the MEC-dentate gyrus pathway may dominate to process spatial information (Fernández-Ruiz et al., 2021). Our results thus provide a clear example of how neuropeptides can act on specific neural circuits to regulate complex behaviors.

While we recognize that inter-male aggression and associated stress in adult C57BL/6 mice may introduce potential confounding effects on social memory readouts (Lidster et al., 2019), we implemented experimental controls including the full physical separation of subject and stimulus mice via wire-mesh cups during testing to eliminate all direct aggressive contact, alongside standardized pre-experimental gentle handling to minimize procedural stress responses. The preserved spatial memory performance in OXTR antagonist-injected mice further confirms that the observed selective social memory encoding phenotype is unlikely to be driven by non-specific stress. Future follow-up work will be dedicated to systematically exploring this potential confounding factor in a more focused experimental design.

To fully elucidate the role of OXT in social memory, several key questions remain to be addressed in future studies. First, *in vivo* recordings of neural oscillations in the LEC and hippocampus during social memory tasks are needed to directly test whether OXT enhances gamma synchrony between these regions. Second, dissecting the relative contributions of the direct and indirect LEC-CA2 pathways to OXT-mediated social memory enhancement will require pathway-specific manipulation techniques, such as retrograde viral tracing combined with optogenetics. Third, the current data cannot fully exclude potential modulatory effects of OXT on other LEC cell type. Future work will conduct systematic population-scale patch-clamp recordings across all morphologically and molecularly defined LEC cell classes, to map the full cell-type-specific OXTR response landscape of this region. By addressing these questions, future research will not only advance our understanding of OXT’s role in social memory but also provide potential targets for treating social cognitive deficits associated with neuropsychiatric disorders such as ASD or schizophrenia.

## Data Availability

The original contributions presented in the study are included in the article/supplementary material, further inquiries can be directed to the corresponding authors.
